# Supplementary material for: Raman time-delay in attosecond transient absorption of strong-field created krypton vacancy
Source: Nat Commun. 2024 Mar 27;15:2705. doi: 10.1038/s41467-024-47088-9 (PMC10973479; doi:10.1038/s41467-024-47088-9)
Supplement: Supplementary file 1 — Supplementary Information [file 41467_2024_47088_MOESM1_ESM.pdf]

Supplementary materials:

Raman time-delay in attosecond transient absorption of strong-field created krypton vacancy

Li Wang[1, 2, §], Guangru Bai [1, 2, §], Xiaowei Wang[1, 2, *], Jing Zhao[1, 2, *], Cheng Gao[1, 2], Jiacan Wang[1, 2], Fan Xiao[1, 2], Wenkai Tao[1, 2], Pan Song[1], Qianyu Qiu[1, 2], Jinlei Liu[1, 2], and Zengxiu Zhao[1, 2, *]

[1]*Department of Physics, National University of Defense Technology, Changsha 410073, China.*

[2]*Hunan Key Laboratory of Extreme Matter and Applications, National University of Defense Technology, Changsha 410073, China*

[§]*These authors contribute equally to this work.*

[*]*Corresponding authors: xiaowei.wang@nudt.edu.cn; jzhao@nudt.edu.cn; zhaozengxiu@nudt.edu.cn.*

Supplementary Note 1: Experimental Details

The schematic drawing for the experimental system is shown in Fig S1. The laser for the experiments was a 1 KHz multi-pass chirped pulse amplification system (Femtopower HE), which delivers 25 fs, 4.2 mJ pulses. The multi-cycle pulses were then coupled to a 1-m long helium-filled hollow-core fiber (HF) with diameter of 300 μm. To decrease the ionization effect and improve the throughput of the fiber, differential pumping scheme was employed. High pressure helium gas (2000 mbar) was introduced from the exit of the fiber, while pressure of the entrance was kept as low as 3E-2 mbar with a dry pump (TriScroll 300). The spectrum was then broadened to cover a spectral range of 580-940 nm, as shown in Fig S2(b). To get ultrashort few-cycle pulses, chirped mirrors (Laser Quantum DCM7) were used for group delay dispersion compensation. After the chirped mirrors, the NIR pulse was then compressed down to 5.3 fs, which was measured with a fringe resolved autocorrelator (Femtometer), as shown in Fig. S2(a). The FWHM of the autocorrelation signal is 10 fs, so the duration of NIR pulses is estimated to be 5.3 fs assuming sech2 temporal shape.

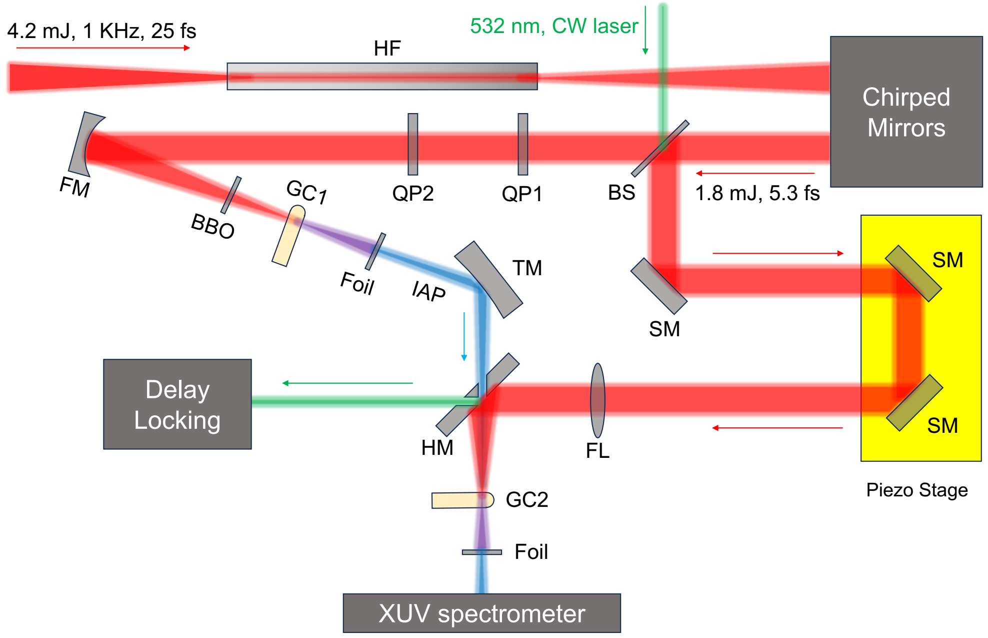

Fig. S1 Detailed experimental layout. HF: 1-m long hollow-core fiber; BS: 50:50 ultrafast beam splitter; QP1: 177 µm thick quartz plate; QP2: 440 µm thick quartz plate; BBO: 141 µm thick BBO crystal; FM: silver-coated focal mirror; GC1: the first gas cell for IAP generation; Foil: metal foil for residual NIR blocking; TM: toroidal mirror; HM: hole mirror; SM: flat silver mirror; FL: focal lens, GC2: the 2nd gas cell for absorption measurements.

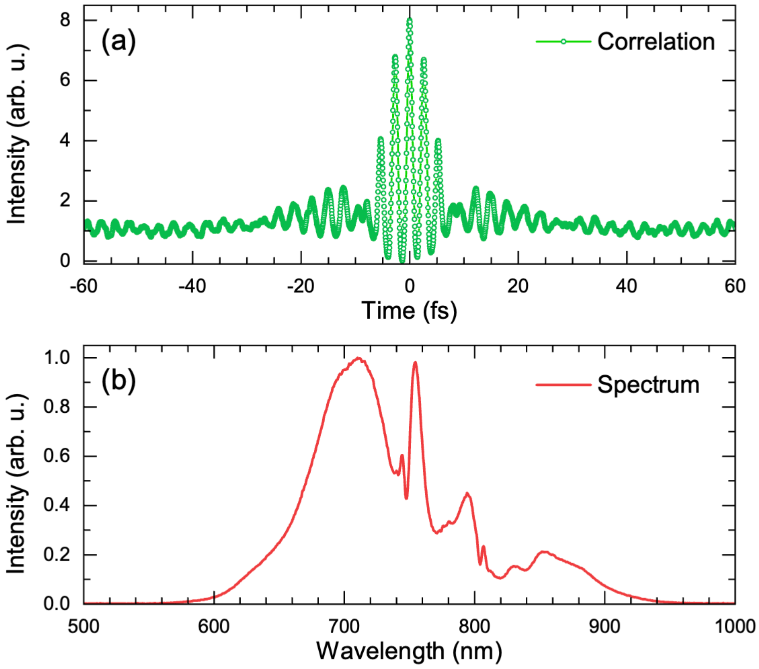

Fig. S2 The duration and spectrum of the ultrashort laser pulse. (a) The fringe resolved autocorrelation signal of the compressed few-cycle pulses after chirped mirrors. The FWHM of the autocorrelation signal is 10 fs, so the duration of NIR pulses is estimated to be 5.3 fs assuming sech2 temporal shape. (b) the spectrum of the broadened pulses after hollow-core fiber.

The few-cycle pulses after the chirp mirrors with pulse energy of 1.8 mJ were divided equally into two parts by a 50% beam splitter (FemtoOptics OA037). The electric field of one arm was manipulated to form a half laser cycle gate for isolated attosecond pulse (IAP) generation with double optical gating (DOG) optics, which consisted of a 177 μm thick quartz plate (QP1), a 440 μm thick quartz plate (QP2) and a 141 μm thick beta-barium borate (BBO) crystal. The pulses were then focused into neon gas filled cell by a concave mirror with focal length of 350 mm to generate IAP. A 200 nm thickness Zr foil (Lebow) was used to block residual NIR beam. The IAP was then focused by a gold coated toroidal mirror (CLaser) into the second gas cell filled with Kr samples (50 mbar). The other arm from the beam splitter was propagated along a delay line installed on a nano-precision piezo-stage (Physik Instrumente, P-752.1CD) and combined with IAP by a hole-drilled mirror (HM). The pump beam was focused by a lens with focal length of 400 mm to generate vacancy in Kr valance shell. The IAP served as the probe of the vacancy generation dynamics. After interacting with Kr gas, the residual NIR was filtered out again using a 200 nm thickness Zr foil (Lebow), and the transmitted IAP was recorded by a home-made XUV spectrometer (see

Supplementary Note 2 for the XUV spectrometer details). The time delay between the NIR and the IAP was stabilized and controlled with active phase locking technology, to implement which a green continuous-wave (cw) laser beam was sent through the two arms of the pump-probe system and formed interference fringes after the HM. The piezo-stage was adjusted by a proportional-integral-differential (PID) control procedure according to the movement of the interference fringe (see Supplementary Note 3 for the delay stabilization details).

The pointing stability of the NIR laser pulses was measured by monitoring the focal spot position with a charge-coupled device (CCD) over 120 minutes, as shown in Fig. S3 (a). The pointing stability was better than 20 μrad. The power stability after hollow-core fiber was measured with a power-meter (Thorlabs PM100D), as shown in Fig. S3(b). The root-mean-square (rms) error of the pulse energy is estimated to be 1.1%.

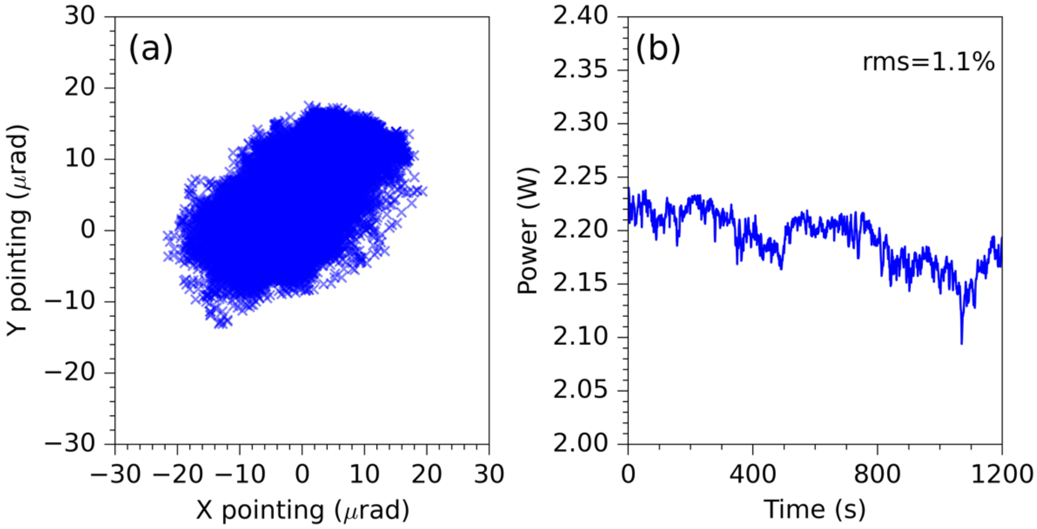

Fig. S3 The stability of the laser. The NIR laser pointing stability (a) and power stability (b). The pointing stability was better than 20 μrad, while the rms error of the pulse energy is estimated to be 1.1%.

Since the wavefront of the IAP pulses is determined by that of the NIR pulses, the IAPs have the same pointing stability as the NIR pulses.

Supplementary Note 2: the XUV spectrometer

The XUV spectrometer used in the experiments[1] consisted of a flat-field grating (Hitachi 001-0660) and a microchannel plate detector (Tectra), as shown in Fig. S4(a). The transmitted XUV beam after the gas cell illuminated the grating with a grazing angle of $\alpha = 87°$, and the diffraction angle β satisfies:

$$sin\alpha - sin\beta = m\lambda G, \tag{R1}$$

where $m = 1$, λ, and $G = 1200\ \mathrm{mm}^{-1}$ are the diffraction order, wavelength and grating constant, respectively. Due to specially designed variable groove space and surface curvature, the spectral components with wavelength between 5-25 nm are focused on a flat focal plane located $L = 563.2\ \mathrm{mm}$ away from the grating. The wavelength of the diffracted spectral component located at position y on the focal plane can be written as:

$$\lambda = \frac{1}{mG}\left(sin\alpha - \sqrt{\frac{L^2}{y^2+L^2}}\right). \tag{R2}$$

It suggests a very complex dependence of wavelength λ on position y. However, it can be approximated by a 3rd order polynomial, as shown in Fig. S4(b). Therefore, the spectrometer was calibrated by fitting some known spectral lines with a 3rd order polynomial.

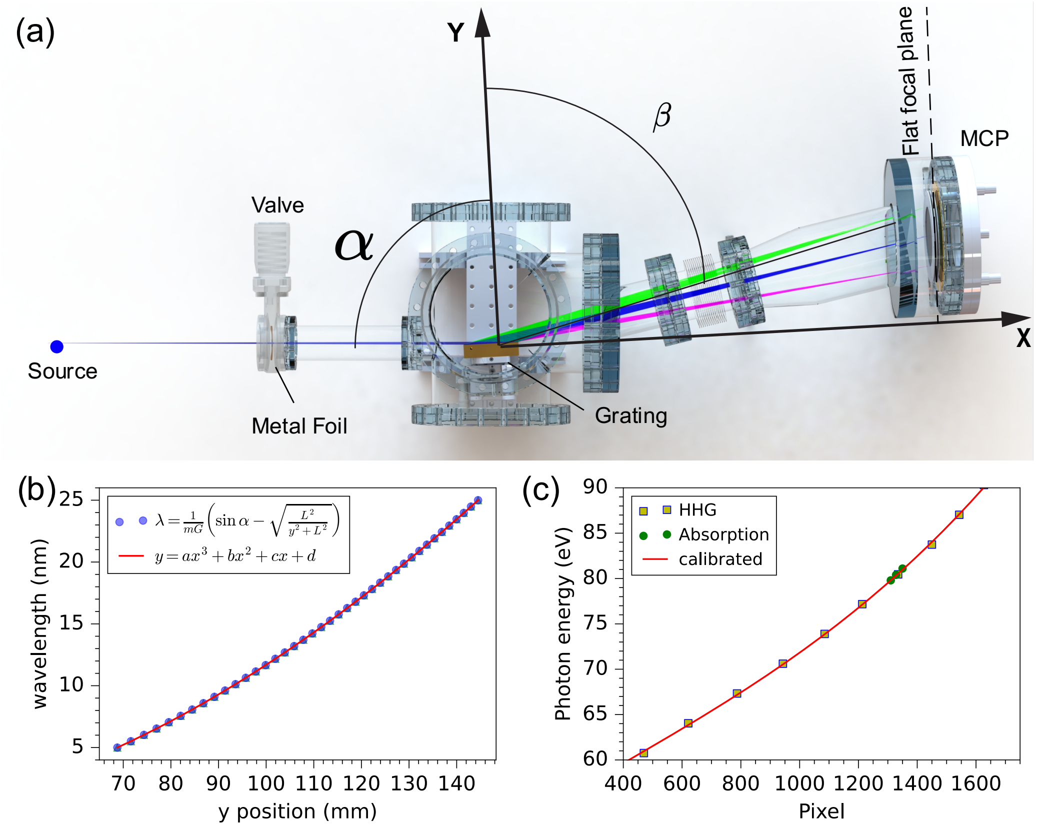

Fig. S4 The schematic and calibration of the XUV spectrometer. (a) The XUV light from the source, i.e., the absorption cell in our experiments, is diffracted by a flat-field grating (Hitach 001-0660) with incident angle of $\alpha = 87°$, forming a flat focal plane located 563.2 mm away from the grating. The MCP detector is placed right on the focal plane to record the spectrum. The Zr foil, which is used to remove NIR laser pulses, is mounted on the center of a vacuum valve installed on the entrance of the spectrometer. (b)

The dependence of diffracted spectral component wavelength on position y on the focal plane can be described by a 3rd order polynomial. (c) The spectrometer was calibrated with three transition lines located at 79.8 eV, 80.4 eV and 81.1 eV (circles), together with discrete harmonic lines (squares).

The three transitions $4p_{3/2}^{-1} \rightarrow 3d_{5/2}^{-1}$, $4p_{1/2}^{-1} \rightarrow 3d_{3/2}^{-1}$ and $4p_{3/2}^{-1} \rightarrow 3d_{3/2}^{-1}$ located at 79.8 eV, 80.4 eV and 81.1 eV respectively were observable on the spectrometer, as shown as the filled circles in Fig. S4(c). Besides, discrete harmonics (squares in Fig. S4(c)) generated with linear polarized long pulses were used for calibration as well. All the spectral lines were then fitted with a 3rd order polynomial as explained before. Note that the fitting was done to convert pixel number to wavelength, then photon energy was obtained from wavelength.

Supplementary Note 3: the stabilization of pump-probe delay

The pump-probe delay control, which is the key to ensure the accurate temporal resolution, was realized with active delay control technique[2]. A continuous green beam was split by the same beam splitter in ATAS setup, so that the two green beams went through the XUV and NIR path, and recombined on the hole-mirror to form interference fringes. By stabilizing the fringes with PID feedback electronics, the pump-probe delay can be stabilized. In Fig. S5(a), we show an example delay scanning fringes which last for 856 seconds. The pump-probe delay can be obtained from the phase of the fringes via $\tau = \frac{\phi T_0}{2\pi}$, where $T_0 = 1.77$ fs is the period of 532 nm green light used in the delay locking setup. The phase of the interference fringes is shown in Fig. S5(b). It is shown that the phase changes 0.5 rad per scan step, i.e., the delay scan step is 141 as. To evaluate the time jitter, we should pay attention to the relative phase change, i.e., the error between the actual phase and target phase, as shown in Fig. S5(c). The rms phase error is 0.08 rad, which suggests a time jitter of 23.8 as.

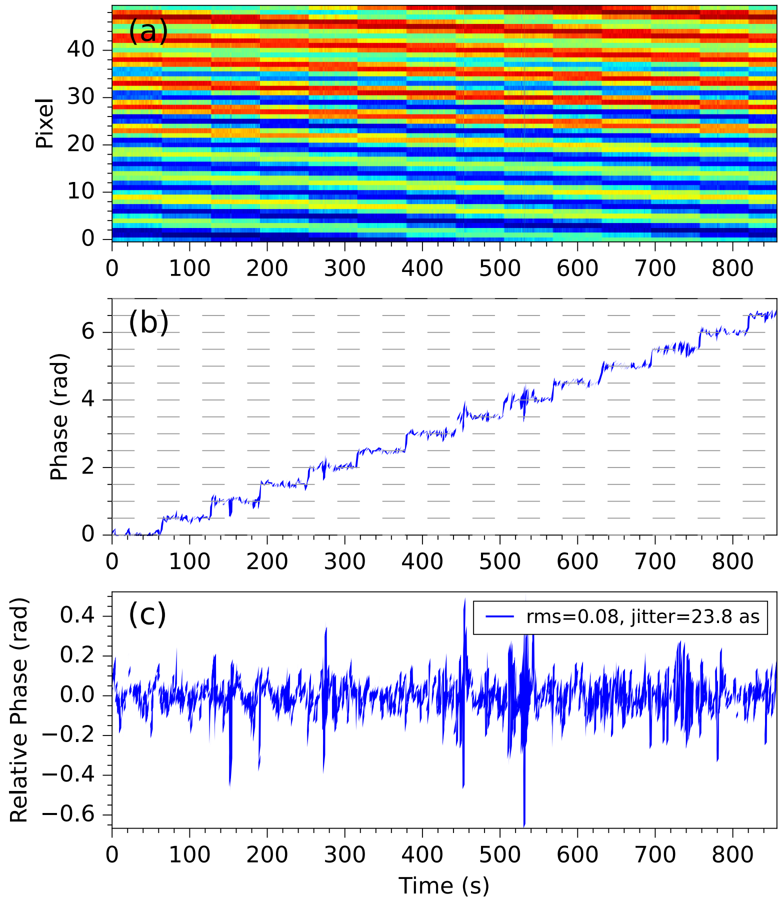

Fig. S5 Active delay stabilization. (a) Interference fringes recorded over 856 seconds when the pump-probe delay is stabilized and scanned. (b) The phase of the fringes extracted from (a) for each delay time. The step-like phase change reflects the pump-probe delay adjustment with scanning step of 0.5 rad (141 as). (c) The relative phase, i.e., the error between actual phase and target phase, indicates a rms error of 0.08 rad (23.8 as).

The actual time resolution of the electron dynamics extracted from ATAS spectra can be much better than either the scan step or delay jitter, since the sinusoidal evolution can be rebuilt with sparse sampling points. For example, 5 as precision was achieved with <30 as jitter and 170 as step size in ATAS measurements previously[3]. By adopting the similar Monte Carlo simulations[4] for RABITT measurements, the temporal resolution of ATAS can also be analyzed. In the simulations shown in Fig. S6(a), we sampled a sinusoidal curve (blue line) with 141 as scan step for different time jitter and scan length, then the sampled points (red circles) were fitted by a sinusoidal curve (red line). The temporal error was obtained by comparing the fitted curve with the true curve. The temporal resolutions, i.e., the statistic error over 20000 simulations, for different time jitter and different scan length are shown in Fig. S6(b). It is shown the precision

becomes better as time jitter decreases and scan length increases. Especially, for 25 as time jitter and 2 optical cycles scan length, the temporal resolution can be as high as 8 as.

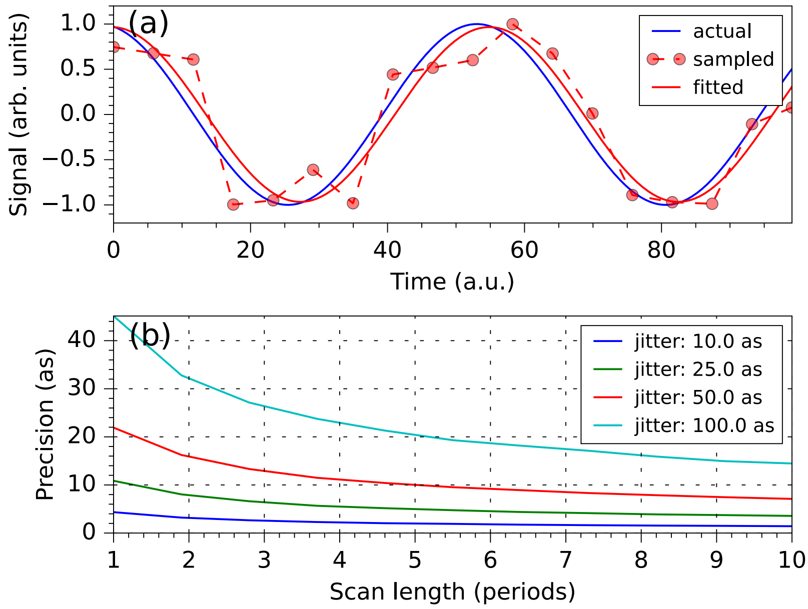

Fig. S6 Temporal resolution analysis for ATAS measurements. (a) a sinusoidal curve (blue line) was sampled with 141 as scan step for a certain time jitter and scan length. The sampled points (red circles) were fitted by a sinusoidal curve (red line). The phase (temporal) difference between the fitted curve and the actual curve gives the temporal precision. (b) The dependence of the precision on time jitter and scan length. Especially, for 25 as time jitter and 2 optical cycles of fundamental pulses, the temporal resolution can be as high as 8 as.

Supplementary Note 4: Experimental raw data and data processing

There are three kinds of spectra measured in the experiments: 1) the spectrum for the input IAP with spectral intensity of S_X^0, 2) the transmitted spectrum after neutral Kr atoms S_X^n and 3) the transmitted spectrum of Kr^+ ion S_X^i. The change of optical density ΔOD is defined as:

$$\Delta OD = -\log\frac{S_x^i}{S_x^0} + \log\frac{S_x^n}{S_x^0} = -\log\frac{S_x^i}{S_x^n} \tag{S1}$$

Therefore, the uncertainty of ΔOD can be evaluated as:

$$e_{\Delta OD} = \sqrt{\left(\frac{\partial \Delta OD}{\partial S_x^i}\mathrm{d}S_x^i\right)^2 + \left(\frac{\partial \Delta OD}{\partial S_x^n}\mathrm{d}S_x^n\right)^2} = \sqrt{\left(\frac{\mathrm{d}S_x^i}{S_x^i}\right)^2 + \left(\frac{\mathrm{d}S_x^n}{S_x^n}\right)^2} \tag{S2}$$

where $\mathrm{d}S_x^i$ and $\mathrm{d}S_x^n$ is the uncertainty of measured transmitted spectra after Kr^+ ion and neutral Kr atoms, respectively.

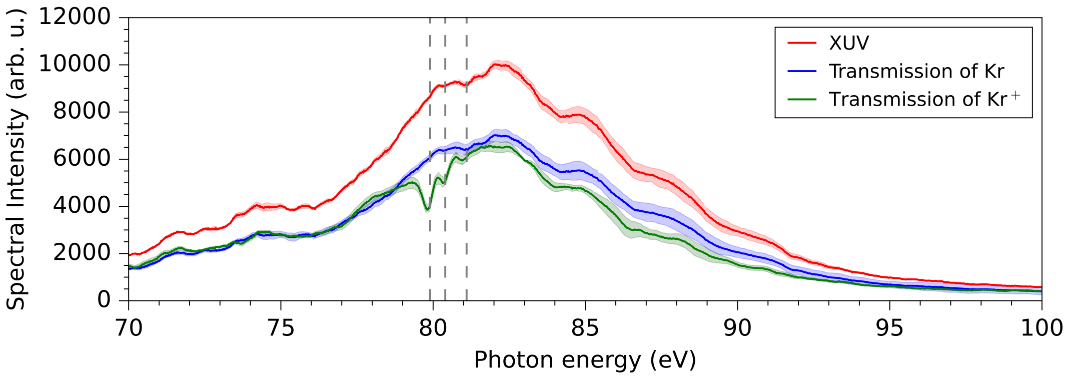

Fig. S7 Typical XUV spectra recorded in the experiments. The input XUV spectrum (red curve), the transmitted spectrum after neutral Kr atoms (blue curve) and the transmitted spectrum after Kr^+ ions (green curve). The filled areas around each curve indicates the statistic intensity uncertainty for each spectrum.

During the acquisition of ATAS traces, each spectrum integrated for 20 s, and the delay scan step size was set to be 141 as with a scan window of 43 fs (307 steps). The entire acquisition lasted for about 108 minutes. Due to laser pointing instability, power fluctuation, thermal expansion of mirror mounts, air turbulence, and any other random changes of the system, the acquired transmission spectra over long time are always noisy, as can be seen from the raw data shown in Fig. S8(a) and (b) for laser intensity of $3.6(\pm0.3)\times10^{14}$ W/cm^2 and $4.6(\pm0.3)\times10^{14}$ W/cm^2, respectively. Therefore, background subtraction is usually needed. The random spectra change occurs in every scan step, manifesting as high frequency oscillations in the ATAS spectrum. It is reasonable to suppress the fast oscillational noise by smoothing the raw data. In the smoothing procedure, each data point is updated by the average of its neighboring data points within a moving window in which Gaussian weights are applied to the data points. By performing the Gaussian-weighted averaging for delay-dependent ΔOD in both the delay axis (3 data points window width) and energy axis (2 data points window width), we obtained the background-free traces, as shown in Fig. S8(c) and (d), respectively. These are the data sets demonstrated in the main manuscript.

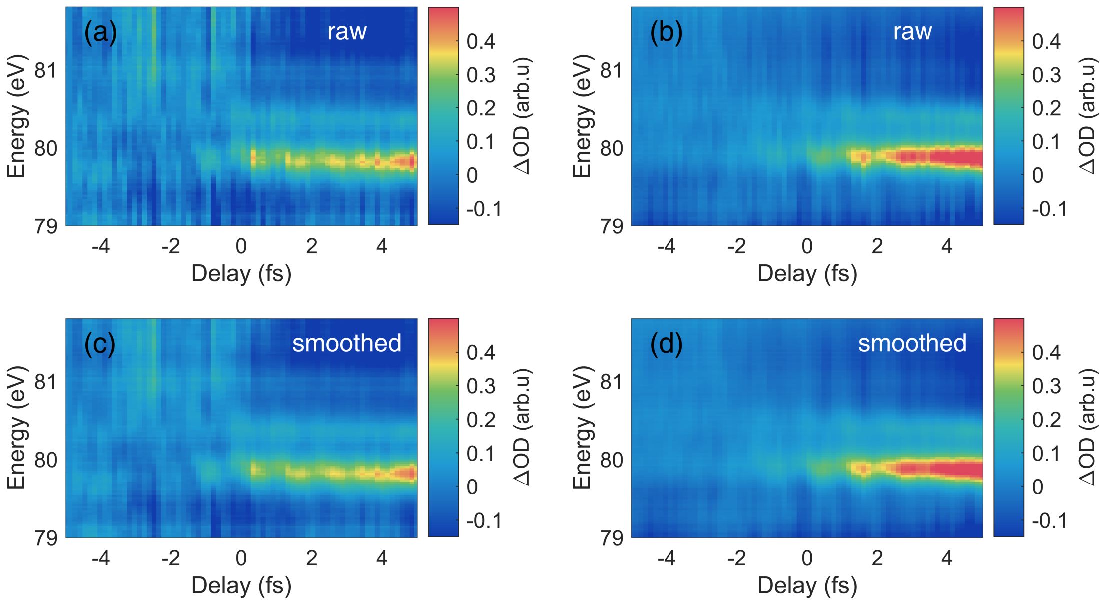

Fig. S8 Raw data in ATAS measurements. The raw data (a,b) and smoothed background-free data (c,d) of the ATAS traces for IR intensity of 3.6(±0.3)×10^{14} W/cm^2 (a,c) and 4.6(±0.3)×10^{14} W/cm^2 (b,d). The smoothing was done with Gaussian-weighted moving average method with window with of 2 data points and 3 data points for the energy axis and delay axis, respectively.

Except for the demonstrated two datasets, the experiments were carried out for multiple times, and the observed dynamics were reproducible. Figure S9(a), (b), (c) and (d) show four additional datasets of ΔOD, in which the oscillations in the absorption of two transition channels and their relative delay are evident.

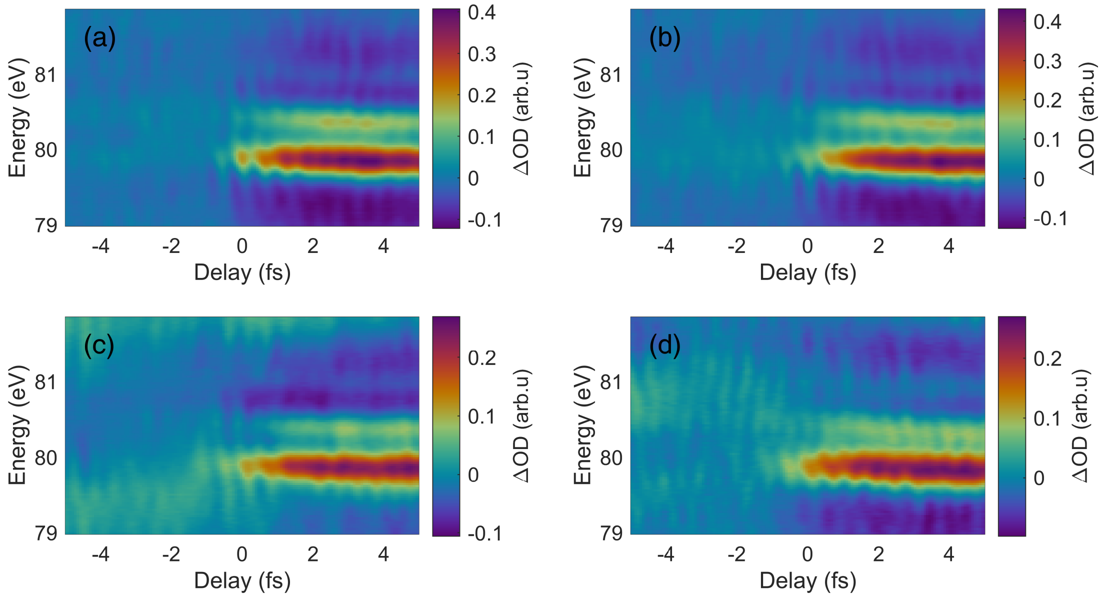

Fig. S9 Four additional datasets for ATAS measurements. The four datasets (a), (b), (c) and (d) were all measured with laser intensity of 3.6(±0.3)×10^{14} W/cm^2. The oscillations in the absorption of two transition channels and their relative delay are evident, showing good data reproducibility.

Supplementary Note 5: the calculation of kr$^+$ ion parameters

The atomic structure parameters such as energy level and transition dipole moment are calculated via the Flexible atomic code (FAC)[5]. The results are summarized in Table S1 and Table S2.

Table S1: Energy levels of Kr$^+$

label	Abridged Notation	Configuration	*J*	Energy (eV)
**1**	$[4p_{3/2}]^{-1}$	$[Ar]3d^{10}4s^24p^5$	1.5	$E_1 = 0.000$
**2**	$[4p_{1/2}]^{-1}$	$[Ar]3d^{10}4s^24p^5$	0.5	$E_1 = 0.666$
**3**	$[4s_{1/2}]^{-1}$	$[Ar]3d^{10}4s^14p^6$	1.5	$E_1 = 13.51$
**4**	$[3d_{5/2}]^{-1}$	$[Ar]3d^94s^24p^6$	0.5	$E_1 = 79.12$
**5**	$[3d_{3/2}]^{-1}$	$[Ar]3d^94s^24p^6$	0.5	$E_1 = 80.32$

Table S2: Transition energies and transition dipole moments

Transition	$\boldsymbol{\Delta E\ (eV)}$	Reduced transition dipole moments (a.u.)
$[\boldsymbol{4p_{3/2}}]^{-1} \rightarrow [\boldsymbol{4s_{1/2}}]^{-1}$	13.51	-2.631
$[\boldsymbol{4p_{1/2}}]^{-1} \rightarrow [\boldsymbol{4s_{1/2}}]^{-1}$	12.844	-1.8558
$[\boldsymbol{4p_{3/2}}]^{-1} \rightarrow [\boldsymbol{3d_{5/2}}]^{-1}$	79.12	-0.4949
$[\boldsymbol{4p_{3/2}}]^{-1} \rightarrow [\boldsymbol{3d_{3/2}}]^{-1}$	80.32	-0.161
$[\boldsymbol{4p_{1/2}}]^{-1} \rightarrow [\boldsymbol{3d_{3/2}}]^{-1}$	76.654	-0.379

The transition dipole moments involving magnetic quantum numbers are obtained by Eq. (S3) according to Wigner-Eckart theorem[6]:

$$\left\langle \gamma JM \middle| T_q^{(k)} \middle| \gamma' J' M' \right\rangle = (-1)^{J-M} \begin{pmatrix} J & k & J' \\ -M & q & M' \end{pmatrix} \langle \gamma J || T^{(k)} || \gamma' J' \rangle \qquad \text{(S3)}$$

where J and J' represent the total angular momentum quantum numbers, M and M' are the total magnetic quantum numbers (M_j notation is used in the paper), γ and γ' are the other quantum numbers, $T_q^{(k)}$ is the q component of a k order irreducible tensor operator $T^{(k)}$, and $\langle \gamma J || T^{(k)} || \gamma' J' \rangle$ is the reduced matrix element which does not depend on M or M'. In the case that NIR and XUV are both linearly polarized in parallel, we have $k = 1$, $q = 0$ and $\Delta M = M - M' = 0$. We consider only $M = \pm 1/2$ because the ionization rates of quantum states with other magnetic quantum numbers are much lower. Note that the quantum states corresponding to $M = -1/2$ and $M = 1/2$ are symmetric, it is enough to carry out calculation only for the case of $M = -1/2$. The corresponding parameters are shown in Table S3.

Table S3: The transition energy and transition dipole moments of Kr^{+} ($M = -1/2$)

**Transition**	$\boldsymbol{\Delta E\ (eV)}$	**transition dipole moments (a.u.)**
$\left[4p_{3/2}^{-1/2}\right]^{-1} \rightarrow \left[4s_{1/2}^{-1/2}\right]^{-1}$	13.51	$d_{13} = -1.07410$
$\left[4p_{1/2}^{-1/2}\right]^{-1} \rightarrow \left[4s_{1/2}^{-1/2}\right]^{-1}$	12.844	$d_{23} = 0.757627$
$\left[4p_{3/2}^{-1/2}\right]^{-1} \rightarrow \left[3d_{5/2}^{-1/2}\right]^{-1}$	79.12	$d_{14} = -0.156501$
$\left[4p_{3/2}^{-1/2}\right]^{-1} \rightarrow \left[3d_{3/2}^{-1/2}\right]^{-1}$	80.32	$d_{15} = 0.020785$
$\left[4p_{1/2}^{-1/2}\right]^{-1} \rightarrow \left[3d_{3/2}^{-1/2}\right]^{-1}$	76.654	$d_{25} = -0.154726$

In order to achieve better agreement with the experiment, the transition dipole moment of $\left[4\boldsymbol{p}_{1/2}^{-1/2}\right]^{-1} \rightarrow \left[4\boldsymbol{s}_{1/2}^{-1/2}\right]^{-1}$ is chosen to be -0.757627 which differs in sign from the structural calculation.

Supplementary Note 6: Computation of strong field ionization rates

The ionization rate of Kr atom can be estimated by the modified ADK formula[7]:

$$W_{ADK}(t) = \frac{C_l^2}{2^{|m_l|}|m_l|!} \frac{(2l+1)(l+|m_l|)!}{2(l-|m_l|)!} \frac{1}{\kappa^{\frac{2Z_c}{\kappa}-1}} \left(\frac{2\kappa^3}{|E_{IR}(t)|}\right)^{\frac{2Z_c}{\kappa}-|m_l|-1} e^{-\frac{2\kappa^3}{3|E_{IR}(t)|}}$$

$$W_m(t) = W_{ADK}(t) e^{-\alpha (Z_C^2/I_p)(|E_{IR}(t)|/\kappa^3)}, \tag{S4}$$

where $\kappa = \sqrt{2I_p}$ with I_p being the lowest ionization potential, $C_l = 2.49$[8], $\alpha = 9$, $z_C = 1$, $l = 1$, $m_l = 0$, and $I_p = 14$ eV.

As the transient ionization occurs within the laser field, the quantum coherence between the continuum and the ground state leads to the back-forth jumping of the population on the continuum, which gives rise to temporal modulation of the ionization rate. Based on strong field approximation (SFA), the time-dependent wave function can be approximated as:

$$\psi(t) \approx \psi_g + \sum_k a_k(t)|k,t\rangle, \tag{S5}$$

where ψ_g is the electronic ground state, and $|k,t\rangle$ represents a plane wave with canonical momentum k moving in the laser field. The ionization probability can be obtained by integration of the continuum population:

$$P(t) = \sum_k |a_k(t)|^2 \tag{S6}$$

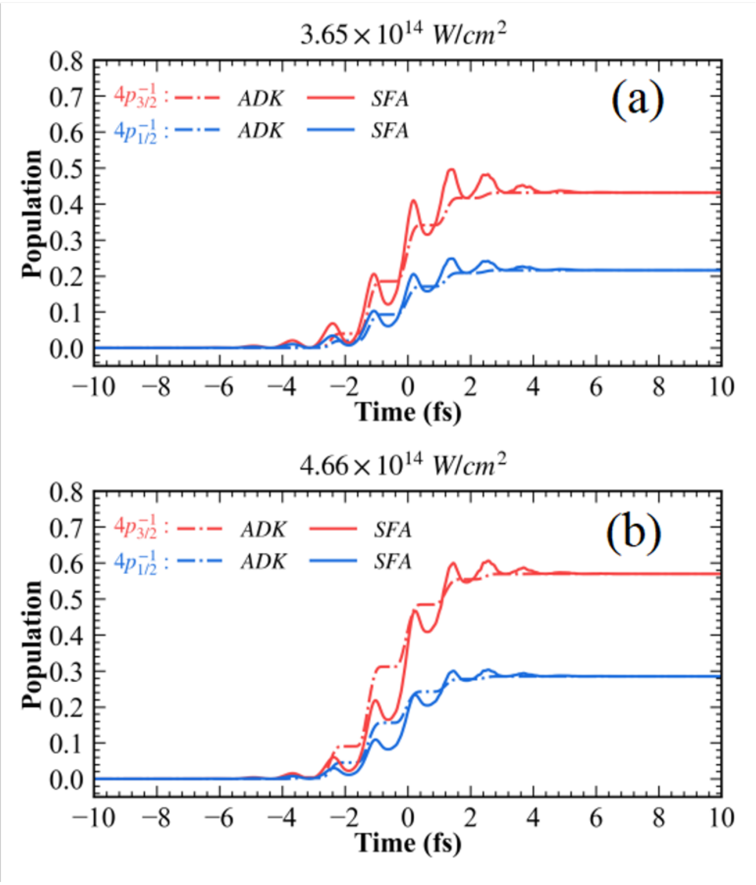

Fig. S10 Temporal population of the ion. Temporal population of the ion

predicted by ADK theory and SFA model for laser intensities of (a) 3.6×10^{14}W/cm^2 and (b) 4.6×10^{14}W/cm^2.

Once the ionization probability is obtained, the corresponding ionization rate can be computed. In order to compare with ADK theory, the final ionization probability from SFA is normalized by ADK results. Figure S10 presents the ionic population due to transit ionization given by the two models. Oscillation of the ionic population can be clearly seen for the SFA model.

Supplementary Note 7: The absorption of Krypton vacancy

Once Eq. (S3) is solved, the induced dipole moment can be obtained as:

$$d(t,t_d) = Tr[\mu\rho^+(t)], \tag{S7}$$

where μ is the transition dipole matrix.

The absorption cross-section can be computed by Eq. (S8):

$$\sigma(\omega,t_d) = \frac{\omega}{c\varepsilon_0} Im\left[\frac{\tilde{d}(\omega,t_d)}{\tilde{\varepsilon}(\omega)}\right], \tag{S8}$$

where ω is the photon angular frequency, c is the speed of light in vacuum, ε_0 is the vacuum permittivity, $\tilde{\varepsilon}(\omega)$ is the Fourier transform of $\varepsilon(t)$ and $\tilde{d}(\omega,t_d)$ is the Fourier transform of $d(t,t_d)$. The comparison of the integrated absorbance with experiment is carried out in the energy interval of 79-79.5 eV and 79.5-80.1 eV respectively.

For completeness, we present the absorption during and after the turn-off of the NIR pulse. Figure S11 shows the calculated absorption of the three resonant transitions at the laser intensity of 4.6×10^{14}W/cm^2, where the coupling with $4s$ is not included. The absorbance of the two transitions involving of $3d_{3/2}^{-1}$ exhibit clear modulation with period of 6.2 fs corresponding to the energy splitting of $4p_{3/2}^{-1}$and $4p_{1/2}^{-1}$[9].

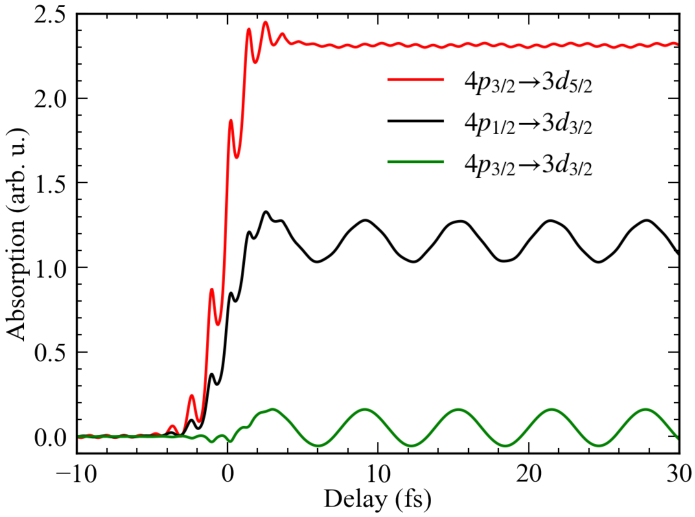

Fig. S11 Numerical results for time-resolved absorption without Raman coupling. Time-resolved absorption for the three transitions: $\left[\mathbf{4}p_{3/2}^{-1/2}\right]^{-\mathbf{1}} \longrightarrow \left[3d_{3/2}^{-1/2}\right]^{-\mathbf{1}}$ (red), $\left[\mathbf{4}p_{3/2}^{-1/2}\right]^{-\mathbf{1}} \longrightarrow \left[3d_{5/2}^{-1/2}\right]^{-\mathbf{1}}$ (black), $\left[\mathbf{4}p_{1/2}^{-1/2}\right]^{-\mathbf{1}} \longrightarrow \left[3d_{3/2}^{-1/2}\right]^{-\mathbf{1}}$ (green). The laser intensity is 4.6×10^{14}W/cm^2.

When the Raman coupling is taken into account, the residual coherence is reduced and thus the modulation depth of the absorbance is less as shown in Fig. S12. Note that there exists emission for the transition $\left[\mathbf{4}p_{3/2}^{-1/2}\right]^{-\mathbf{1}} \longrightarrow \left[3d_{3/2}^{-1/2}\right]^{-\mathbf{1}}$ at the near zero negative time-delay.

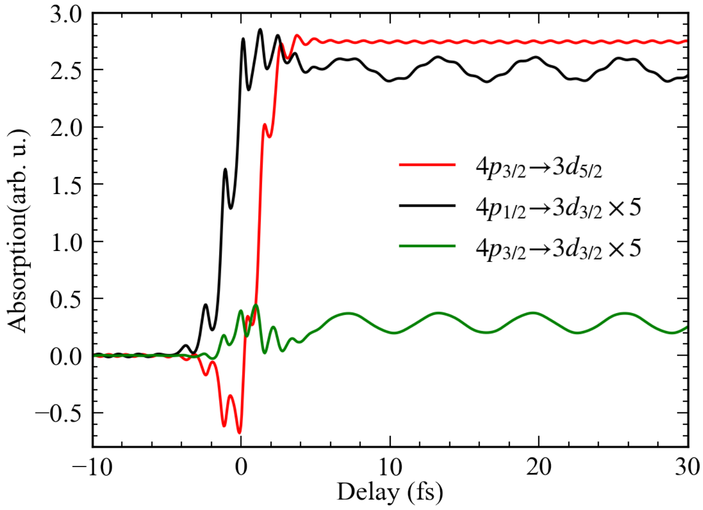

Fig. S12 Numerical results for time-resolved absorption with Raman coupling. Same as Fig S11, but with Raman coupling with $4s$ orbital

included.

Supplementary Note 8: Calculations with more coupling states

In order to corroborate our analysis, we performed calculations with 11 essential multi-configuration states (see Table S4), and resulted in slightly increased time delays (240 as), as shown in Fig. S13(d). The Raman effect can be pinpointed by looking into the instantaneous population and delay-dependent phase shift. It is known that the absorption profile is determined by oscillation strength and the phase shift of the dipole as described by the Eq. (17) in Ref[10]. The modulation of the absorption strength at the resonant energy can be written as $\rho_{\mathrm{i}}\cos[\phi_i(\tau)]$. The phase shift can be retrieved from the simulation by taking the Fourier transform of the induced dipole, i.e. $\phi = \mathrm{Arg}[d(\omega)]$ for the resonant transition energy ω_0. In order to examine the origin of the phase shift, we calculate the instantaneous eigen energies by diagonalizing the instantaneous Hamiltonian of the 11 coupled states. Under this adiabatic picture, the phase shift can be approximated by $\varphi = \int \Delta E dt$, where ΔE is the adiabatic energy shift. It can be seen in Fig. S13(a) that the phase variations with delay obtained by the two manners are qualitatively consistent. The discrepancy is more evident in $\cos[\phi_i(\tau)]$, as shown in Fig. S13(b). The adiabatic phase shift gives step-like rising, while the calculated phase gives half-cycle modulations arising from non-adiabatic effects. The population redistributed by the Raman coupling are plotted in Fig. S13(c). The modulations of the two population differ by relative time delay of about 40 attoseconds. As both the Raman coupling caused phase shift and population variation contribute to the resonant absorbance, the time delay of the two resonant absorption lines is found to be 240 attoseconds, as indicated in Fig. S13(d). Similar calculations are performed for the laser intensity of I_2 as shown in Fig. S14. Therefore, the multiple state calculation confirms the reliability of our model.

Table S4: Energy levels of Kr+. The energies of the corresponding states are calculated via Multi-Configuration Dirac-Fock method.

**label**	**Configuration**	**J**	**Energy (eV)**	**Comments**
**0**	$[Ar]3d^{10}4s^24p^5$	1.5	0.000	Kr^+ ground state

**1**	$[Ar]3d^{10}4s4p^6$	1.5	13.15	Coupling states considered in our calculations
**2**	$[Ar]3d^{10}4s^24p^44d$	0.5	13.86	
**3**	$[Ar]3d^{10}4s^24p^45s$	0.5	13.97	
**4**	$[Ar]3d^{10}4s^24p^45d$	0.5	21.51	
**5**	$[Ar]3d^{10}4s^24p^45s$	1.5	13.04	
**6**	$[Ar]3d^{10}4s^24p^44d$	1.5	17.57	
**7**	$[Ar]3d^{10}4s^24p^45d$	1.5	19.71	
**8**	$[Ar]3d^{10}4s^24p^45s$	2.5	14.74	
**9**	$[Ar]3d^{10}4s^24p^44d$	2.5	15.84	
**10**	$[Ar]3d^{10}4s^24p^45d$	2.5	19.93	
**11**	$[Ar]3d^{10}4s^24p^46d$	2.5	21.51	

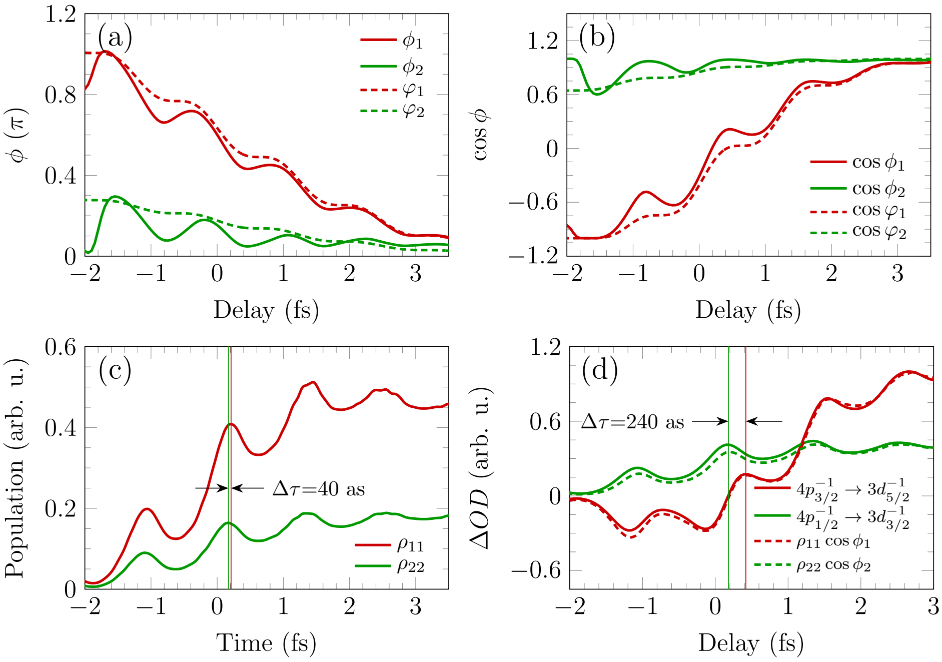

Fig. S13 Calculation results with 11 essential multi-configuration states for the laser intensity I₁. (a) The delay-dependent phase corresponding to the transitions $4p_{3/2}^{-1} \rightarrow 3d_{5/2}^{-1}$ (ϕ_1) and $4p_{1/2}^{-1} \rightarrow 3d_{3/2}^{-1}$($\phi_2$) for the calculation (solid lines) and the adiabatic results (dot dash lines) (φ_1:$4p_{3/2}^{-1} \rightarrow 3d_{5/2}^{-1}$(red); φ_2 :$4p_{1/2}^{-1} \rightarrow 3d_{3/2}^{-1}$(green)). (b) The cosine function corresponding to the calculated phase (solid lines) ϕ_1 (red) and ϕ_1 (green) and the adiabatic phase (dot dash lines) φ_1 (red) and φ_2 (green). (c) The

time-dependent population of the two ionic states $4p_{3/2}^{-1}$ (red) and $4p_{1/2}^{-1}$ (green). (d) The absorption of the transitions $4p_{3/2}^{-1}$⟶$3d_{5/2}^{-1}$ (red) and $4p_{1/2}^{-1}$⟶$3d_{3/2}^{-1}$ (green) at their resonant energy in our calculation (solid lines) and the corresponding analytical results (dotted lines) obtained via $\rho_{\mathrm{i}}\cos[\phi_i(\tau)]$.

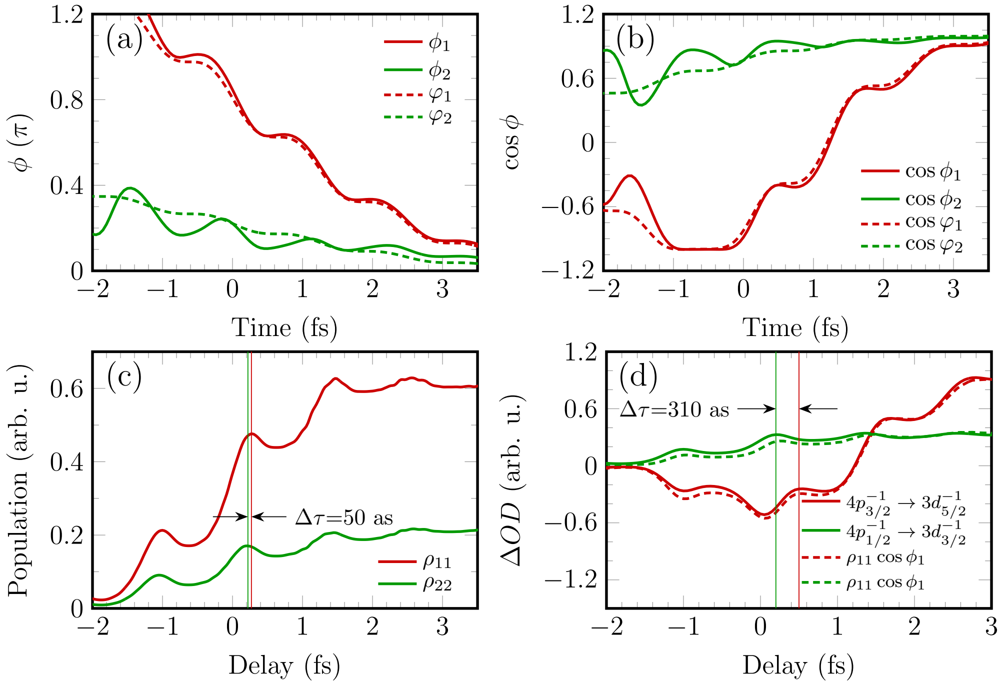

Fig.S14 Calculation results with 11 essential multi-configuration states for the laser intensity I₂. (a) The delay-dependent phase corresponding to the transitions $4p_{3/2}^{-1}$⟶$3d_{5/2}^{-1}$ (ϕ_1) and $4p_{1/2}^{-1}$⟶$3d_{3/2}^{-1}$(ϕ_2) for the calculation (solid lines) and the adiabatic results (dot dash lines) (φ_1:$4p_{3/2}^{-1}$ ⟶ $3d_{5/2}^{-1}$(red); φ_2 :$4p_{1/2}^{-1}$ ⟶ $3d_{3/2}^{-1}$(green)). (b) The cosine function corresponding to the calculated phase (solid lines) ϕ_1 (red) and ϕ_1 (green) and the adiabatic phase (dot dash lines) φ_1 (red) and φ_2 (green). (c) The time-dependent population of the two ionic states $4p_{3/2}^{-1}$ (red) and $4p_{1/2}^{-1}$ (green). (d) The absorption of the transitions $4p_{3/2}^{-1}$⟶$3d_{5/2}^{-1}$ (red) and $4p_{1/2}^{-1}$⟶$3d_{3/2}^{-1}$ (green) at their resonant energy in our calculation (solid lines) and

the corresponding analytical results (dotted lines) obtained via $\rho_{\mathrm{i}}\cos[\phi_i(\tau)]$.

References:

1. Wang, X., Chini, M., Cheng, Y., Wu, Y. & Chang, Z. In situ calibration of an extreme ultraviolet spectrometer for attosecond transient absorption experiments. *Applied Optics* **52**, 323–329 (2013).
2. Chini, M. *et al.* Delay control in attosecond pump-probe experiments. *Optics Express* **17**, 21459 (2009).
3. Hartmann, M. *et al.* Attosecond precision in delay measurements using transient absorption spectroscopy. *Optics Letters* **44**, 4749 (2019).
4. Isinger, M. *et al.* Accuracy and precision of the RABBIT technique. *Philosophical Transactions of the Royal Society A: Mathematical, Physical and Engineering Sciences* **377**, 20170475 (2019).
5. Gu, M. F. The flexible atomic code. *Can. J. Phys.* **86**, 675–689 (2008).
6. Cowan, R. D. *The Theory of Atomic Structure and Spectra*. (University of California Press, 1981). doi:10.1525/9780520906150.
7. Tong, X. M. & Lin, C. D. Empirical formula for static field ionization rates of atoms and molecules by lasers in the barrier-suppression regime. *Journal of Physics B: Atomic, Molecular and Optical Physics* **38**, 2593–2600 (2005).
8. Tong, X. M., Zhao, Z. X. & Lin, C. D. Theory of molecular tunneling ionization. *Physical Review A - Atomic, Molecular, and Optical Physics* **66**, 11 (2002).
9. Santra, R., Yakovlev, V. S., Pfeifer, T. & Loh, Z.-H. Theory of attosecond transient

absorption spectroscopy of strong-field-generated ions. *Phys. Rev. A* **83**, 033405 (2011).

10. Pabst, S. *et al.* Theory of attosecond transient-absorption spectroscopy of krypton for overlapping pump and probe pulses. *Phys. Rev. A* **86**, 063411 (2012).
